# Supplementary material for: DNA barcoding continues to identify endangered species of shark sold as food in a globally significant shark fin trade hub
Source: PeerJ. 2024 Jan 3;12:e16647. doi: 10.7717/peerj.16647 (PMC10771092; doi:10.7717/peerj.16647)
Supplement: Supplemental Information 1 — Examples of DNA sequences used in this work. Note for each species, only a single haplotype was found in all instances. [file peerj-12-16647-s001.docx]

Examples of DNA sequences used in this work. Note for each species, only a single haplotype was found in all instances.

Rhizoprionodon acutus

CCATTTAGCCGGTGTTTCATCAATCTTAGCCTCAATTAACTTTATTACAACCATTATTAATATAAAACCACCAGCCATTTCCCAATACCAAACCCCTTTATTTGTTTGATCTATTCTTGTAACTACTATTCTCCTTCTTCTCTCACTTCCAGTCCTTGCAGCAGGGATTACAATATTACTTACAGATCGCAACCTTAACACCACATTCTTTGATCCTGCAGGTGGGGGAGACCCAATCCTTTACCAACACCTATTCTGATTCTTCGGACA

*Carcharhinus falciformis*

TTAGCTATTTTCTCTCTTCACTTAGCCGGTGTGTCATCTATTCTAGCTTCAATTAATTTTATTACAACTATTATCAATATAAAACCACCAGCCATTTCCCAATATCAAACACCATTATTTGTTTGATCTATTCTTGTAACCACTATTCTTCTCCTCCTATCACTTCCAGTTCTTGCAGCAGGGATTACAATATTACTTACAGATCGTAACCTTAATACTACATTCTTTGATCCTGCAGGTGGAGGAGACCCAATCCTTTATCAACATTTATTTTG

*Galeorhinus galeus*

TGGGACCAATCTGTAGATTTAGCCATTTTCTCCCTTCATTTAGCCGGTATCTCATCAATCCTAGGCTCAATTAATTTTATTACAACCATTATTAACATAAAACCCCCAGCTATTTCCCAATATCAAACACCATTATTTGTTTGATCAATTCTTGTAACTACTATTCTTCTTCTCCTCTCTCTCCCAGTTCTCGCAGCAGGAATCACAATATTACTTACAGACCGTAACCTTAATACCACATTCTTTGACCCTGCAGGTGGAGGAGACCCAATCCTTTACCAACATTTATTCTGATTCTTCGGACATCCAGGAAGTTTA

*Sphyrna lewini*

CTGGGAACCAATCTGTTGACTTAGCTATCTTTTCTCTCCACCTAGCCGGTGTATCATCAATTTTAGCCTCAATTAATTTCATTACAACTATCATTAACATGAAACCTCCAGCCATTTCTCAATATCAAACACCATTATTTGTTTGATCCATCCTTGTAACTACTATCCTACTTCTCCTATCACTTCCAGTTCTTGCAGCAGGAATTACAATATTACTCACAGATCGCAACCTTAATACTACATTCTTTGATCCTGCAGGGGGAGGAGATCCAATCCTTTATCAACACTTATTCTGATTCTTCGGACA

*Sphyrna zygaena*

CCGTTGACTTAGCTATTTTTTCTCTTCATTTAGCTGGTGTATCATCAATCTTAGCCTCAATCAATTTTATTACAACTATTATCAATATAAAACCCCCAGCCATCTCCCAATATCAAACACCACTATTTGTTTGATCTATCCTTGTAACTACTATTTTACTTCTCCTCTCACTTCCAGTTCTTGCAGCAGGGATTACAATATTACTTACAGATCGTAACCTTAATACTACATTCTTTGATCCCGCAGGAGGAGGAGATCCAATTCTTTATCAACACTTATTCTGATT

*Rhizoprionodon oligolinx*

TGTTGACCTAGCTATTTTCTCCCTTCATTTAGCCGGTGTTTCATCAATTTTAGCCTCAATCAATTTTATTACAACTATTATTAATATAAAACCACCAGCCATTTCCCAATACCAAACACCTTTATTTGTCTGATCTATTCTCGTAACTACTATTCTTCTCCTTCTCTCACTTCCAGTTCTTGCAGCAGGAATTACAATATTACTTACAGATCGCAACCTTAATACTACATTCTTTGATCCTGCAGGTGGGGGAGATCCAATCCTTTATCAACATTTATTCTG

*Carcharhinus brevipinna*

TGATTTAGCTATTTTCTCTCTTCACTTAGCCGGTGTTTCATCAATCTTAGCTTCAATTAATTTTATCACAACTATTATTAACATAAAACCACCAGCCATTTCCCAATATCAAACACCATTATTTGTTTGATCTATTCTTGTAACCACTATTCTTCTTCTCCTTTCACTTCCAGTCCTTGCAGCAGGGATTACAATATTACTTACAGATCGTAACCTTAATACTACATTCTTTGACCCTGCAGGCGGAGGAGACCCAATCCTTTATCAACATTTATTCTGAT

*Carcharhinus sorrah*

TCTGTTGATTTAGCTATTTTCTCTCTCCACTTAGCTGGTGTTTCATCAATTTTAGCTTCAATTAATTTTATTACAACTATTATTAATATAAAACCACCAGCCATCTCCCAATATCAAACACCATTATTTGTCTGATCTATTCTTGTAACCACTATTCTCCTTCTCCTCTCACTTCCAGTTCTTGCAGCAGGAATTACAATATTACTTACAGATCGTAACCTTAATACTACATTCTTTGATCCTGCAGGTGGAGGAGATCCAATCCTTTATCAACATTTATTTTG

*Isurus oxyrinchus*

TCTAGCCATTTTTTCCCTCCACCTGGGCTGGTATCTCGTCCATCCTAGCTTCCATTAACTTCATTACAACCATCATCAACATAAAACCCCCAGCAATCTCCCAATACCAAACACCCCTGTTTGTCTGGTCCATTCTAGTGACAACCATCCTCCTTCTTTTAGCACTCCCAGGGCTCGCCGCTGGCATTACAATACTACTTACGGACCGAAACCTAAACACAACATTCTT

*Carcharhinus leucas*

TGGGGCCATCTGTTGACTTAGCTATCTTCTCCCTTCACTTAGCTGGTGTTTCATCAATTTTAGCTTCAATTAATTTTATTACAACTATTATTAATATAAAACCACCAGCCATTTCCCAATATCAAACACCATTATTTGTCTGATCCATTCTTGTAACCACTATTCTTCTTCTCCTTTCACTCCCAGTCCTTGCAGCAGGGATTACAATATTACTTACAGATCGTAACCTTAATACTACATTCTTTGATCCTGCAGGTGGAGGAGACCCAATCCTTTATCAACACTTATTTT

*Carcharhinus melanopterus*

TTTTTCTCTTCACTTAGCTGGTGTTTCATCAATTTTAGCTTCAATTAATTTTATTACAACCATTATTAACATAAAACCACCAGCCATTTCCCAGTATCAAACACCATTATTTGTTTGATCTATTCTTGTAACCACTATTCTACTTCTCCTTTCACTTCCAGTTCTTGCAGCAGGGATTACTATATTACTTACAGATCGTAACCTTAATACTACATTCTTTGATCCTGCAGGGGGAGGAGATCCAATCCTTTATCAACACCTATTTTGATTCTTCGGACA

*Galeocerdo cuvier*

CTGGGACCATCTGTTGATTTAGCAATTTTCTCTCTTCACTTAGCTGGTGTTTCATCAATTTTAGCCTCAATTAACTTTATTACAACTATCATTAATATAAAACCCCCAGCTATCTCCCAATATCAAACACCATTATTTGTATGATCTATTCTTGTTACTACTATTCTCCTTCTTCTTTCACTTCCAGTTCTTGCAGCAGGAATTACAATACTACTTACAGACCGTAACCTTAATACTACATTCTTTGATCCAGCGGGTGGAGGAGATCCAATCCTTTATCAGCACTTATTCTGAT

*Hemipristis elongate*

CTTGGGGCCGTCCGTTGAACTTAGCTATTTTCTCTCTTCATTTAGCTGGGAGTTTCATCAATCTTGGCCTCAATTAACTTTATCACAACTATTATTAATATAAAACCCCCGGCTATCTCTCAATATCAAACACCGCTATTCGTTTGATCAATTCTTGTAACTACTATCCTTCTTCTCCTCTCACTTCCAGTTCTTGCAGCAGGTATTACAATATTACTTACAGATCGTAACCTCAACACTACATTCTTCGACCCTGCAGGGGGAGGTGATCCAATCCTATATCAACACCTATTCTGATTTTTTG

*Triaenodon obesus*

GACCATCTGTTGATTTAGCTATTTTCTCTCTTCACTTAGGCCGGAGTTTCATCAATTTTAGCTTCAATTAATTTTATCACAACTATTATCAATATAAAACCACCAGCCATTTCCCAATATCAAACACCATTATTCGTTTGATCTATTCTTGTAACCACTATTCTTCTTCTCCTTTCACTCCCAGTTCTTGCAGCAGGAATTACAATATTACTTACTGATCGAAACCTTAATACTACATTCTTTGACCCTGCAGGTGGGGGAGACCCAATTCTTTATCAACATTTATTTTGATTCTTCGGACAC

*Carcharhinus macloti*

CCGTCGATTTAGCTATTTTCTCTCTTCACTTAGCCGGTGTTTCATCAATTTTAGCTTCAATTAATTTTATTACAACTATTATTAATATAAAACCACCAGCTATTTCTCAATATCAAACACCATTATTTGTTTGATCTATTCTTGTAACCACTATTCTTCTTCTACTCTCACTTCCAGTTCTTGCAGCAGGAATTACAATATTACTTACAGATCGTAACCTCAATACTACATTCTTTGATCCTGCAGGTGGAGGAGACCCAATCCTTTATCAACATTTATTTT

*Loxodon macrorhinus*

TCTGTTGACCTAGCCATCTTCTCCCTCCATTTAGCTGGTGTTTCATCAATCCTAGCCTCAATTAACTTCATTACAACTATCATTAACATAAAACCACCAGCCATTTCTCAATACCAAACACCATTATTTGTTTGATCAATTCTTGTAACTACTGTCCTTCTTCTCCTCTCACTTCCAGTTCTTGCAGCAGGAATTACAATACTACTTACAGACCGTAACCTTAATACCACATTCTTTGATCCTGCGGGTGGAGGAGACCCAATCCTTTATCAACACTTATTCTG

*Mustelus canis*

GATTTAGCCATCTTCTCCCTTCATTTAGCCGGTATTTCATCAATCTTAGCCTCAATTAACTTTATTACAACTATTATTAATATAAAACCACCAGCCATTTCCCAATATCAAACACCATTATTTGTTTGATCAATTCTCGTGACTACTGTCCTTCTTCTTCTCTCCCTGCCAGTTCTTGCAGCAGGGATTACAATATTACTCACAGACCGAAACCTTAATACTACATTCTTTGACCCCGCTGGGGGAGGGGATCCCATCCTTTACCAACACTTATTCTGATTCTTCGGACACCC

*Eusphyra blochii*

TTGAACCTAGCTATCTTCGTCTCTTCACTTAGCTGGGTGTCTCATCAATCTTAGCCTCAATTAATTTTATCACAACTATTATCAACATAAAACCCCCAGGTATCTCCCAATATCAAACACCCTTATTTGTTTGATCTATCCTTGTAACTACTATTCTACTTCTCCTTTCACTTCCAGTTCTTGCAGCAGGAATTACAATATTACTTACAGACCGCAACCTTAATACTACATTCTTTGATCCTGCAGGAGGAGGAGATCCAATCCTTTATCAACATTTATTCTGATTTTT

*Hemigaleus microstoma*

TTGATTTAGCTATCTTCTCCCTCCATCTAGCTGGTATTTCATCAATTTTAGCCTCAATCAACTTCATCACAACTATTATTAATATAAAACCCCCAGCCATTTCTCAATATCAAACACCATTATTTGTTTGATCCATCCTTGTAACTACTATTCTCCTTCTCCTCTCCCTCCCAGTTCTTGCAGCAGGAATTACTATATTACTTACAGACCGCAACCTTAATACCACATTCTTTGATCCTGCAGGTGGAGGAGACCCAATCCTTTACCAACATCTATTCTG

*Chaenogaleus macrostoma*

GTCGACTTAGCTATTTTCTCCCTCCATTTAGCTGGTATTTCATCAATTTTAGCCTCAATCAACTTCATTACAACTATTATTAATATAAAACCCCCAGCTATTTCTCAATATCAAACACCATTATTCGTTTGATCTATTCTTGTAACTACTATCCTCCTCCTCCTTTCCCTCCCAGTTCTTGCAGCAGGAATTACAATGTTACTTACAGACCGTAACCTTAATACCACATTCTTTGACCCTGCAGGTGGAGGAGACCCAATTCTTTACCAACACCTATTCTGATTCTTCGGAC

*Lamiopsis temminckii*

CCATCTGTTGATTTGGCTATTTTCTCTCTTCACTTAGCTGGTGTCTCATCAATCTTAGCTTCAATCAATTTCATCACAACTATTATTAATATAAAACCACCAGCCATTTCTCAATATCAAACACCATTATTTGTTTGATCCATCCTTGTAACTACTATCCTTCTTCTTCTTTCACTTCCAGTTCTTGCAGCAGGAATTACAATATTACTCACAGACCGTAACCTCAATACTACATTCTTCGACCCTGCAGGTGGAGGAGACCCCATCCTTTATCAACATTTATTTT

*Hemigaleus australiensis*

GTTGATTTAGCTATCTTCTCCCTCCATTTAGCTGGTATTTCATCAATTTTAGCCTCAATCAACTTCATCACAACTATTATTAATATAAAACCCCCAGCCATTTCTCAATATCAAACACCACTATTTGTTTGATCTATCCTTGTAACTACTATTCTTCTCCTCCTTTCCCTCCCAGTTCTTGCAGCAGGAATTACAATATTACTCACAGACCGCAACCTTAATACTACATTCTTTGATCCTGCAGGTGGAGGAGACCCAATCCTTTACCAACACCTATTCTG

*Chiloscyllium punctatum*

ATCAGTTGATTTAACTATTTTCTCCTTACACTTAGCAGGAATTTCATCAATCCTAGCCTCTATCAATTTCATCACAACCATTATCAATATAAAACCACCAGCCATTTCTCAATATCAAACACCCTTATTTGTATGATCAATTCTTGTAACTACCATCCTTCTACTTCTTTCATTACCAGTTCTAGCAGCAGGTATTACTATATTACTTACAGATCGAAACCTAAATACAACATTCTTTGATCCAGCAGGAGGAGGCGATCCTATTTTATATCAACACCTATTCTG

*Prionace glauca*

TTAGCTATTTTCTCTCTTCACTTAGCCGGTATTTCATCAATTTTAGCTTCAATTAACTTTATTACAACCATTATTAATATAAAACCACCAGCCATTTCCCAATATCAAACACCATTATTTGTTTGATCTATTCTTGTAACCACTATTCTTCTTCTCCTAGCACTTCCAGTTCTTGCAGCAGGTATTACAATATTACTTACAGATCGTAACCTTAATACTACATTCTTTGACCCTGCAGGTGGAGGAGATCCAATCCTTTATCAGCACTTATTTTGAT

*Rhizoprionodon taylori*

ATCTGTTGACCTGGCTATTTTCTCCCTCCATTTGGCCGGTGTTTCATCAATTTTAGCCTCAATTAACTTTATTACAACTATCATTAATATAAAACCACCAGCTATTTCCCAATATCAAACACCTTTATTTGTTTGATCTATTCTTGTAACTACTATTCTCCTCCTCCTTTCACTTCCAGTCCTTGCAGCAGGGATTACAATATTACTTACAGACCGTAACCTTAATACCACATTCTTTGATCCTGCGGGTGGGGGAGATCCAATTCTTTACCAACATCTATTC

*Sphyrna mokarran*

CGTTGATCTAGCCATCTTCTCTCTCCACTTAGCTGGTATCTCATCAATCCTGGCCTCAATTAATTTCATCACAACTATTATTAACATAAAACCCCCAGCTATTTCTCAATACCAAACACCATTATTTGTCTGATCTATTCTTGTAACTACTATTCTACTTCTCCTTTCACTTCCAGTCCTTGCAGCAGGAATTACAATATTACTTACAGATCGCAACCTTAATACTACATTCTTTGACCCTGCAGGAGGAGGAGATCCAATTCTTTATCAACATTTATTCTGATTCTTCGGAC
